# Effective Technique Using Combined CO_2_ Laser and Pulsed Dye Laser for Facial Angiofibromas Management in Tuberous Sclerosis

**DOI:** 10.1155/2024/9775613

**Published:** 2024-09-16

**Authors:** Giuseppe Lodi, Giovanni Cannarozzo, Irene Fusco, Tiziano Zingoni, Elena Campione, Mario Sannino

**Affiliations:** ^1^ Unit of Dermatology - University of Campania “Luigi Vanvitelli”, Caserta, Italy; ^2^ Lasers in Dermatology Unit University of Rome Tor Vergata, Rome, Italy; ^3^ El.En. Group, Calenzano 50041, Italy

## Abstract

**Background:**

Facial angiofibromas (FAs) are a dermatological characteristic which are typically linked to tuberous sclerosis (TS).

**Aim:**

We discuss our experience, highlighting a rare occurrence of multiple FAs in a young patient, successfully treated with ablative CO_2_ laser combined with PDL therapy.

**Methods:**

A 23-year-old male patient affected by TS who presents multiple erythematous and colored papules/nodules located on the face, mainly concentrated around nose, perinasal area, cheeks, and chin area, was treated with a combination of ablative CO_2_ laser and a pulsed dye laser. The patient underwent 3 sessions of combined treatment with CO_2_ and pulsed dye laser. The intralesional dye laser treatment was administered immediately after the CO_2_ laser session. The time interval between the combined laser treatments was approximately two months.

**Results:**

After 4 months from the last laser treatment session, most of the facial erythematous and protruding lesions had improved. Following these procedures, the patient did not experience complications or severe adverse reaction.

**Conclusion:**

The combined use of the CO_2_ and dye laser has been proved to be a safe and effective treatment for multiple FAs in the young patient affected by tuberous sclerosis.

## 1. Introduction

Facial angiofibromas (FAs) are a dermatological characteristic which are typically linked to tuberous sclerosis (TS). FAs can have a prevalence of up to 90% [1] and frequently lead patients to contact with specialists about cosmetic procedures to improve their appearance [2].

FAs typically appear in childhood, and the prevalence continues to increase into adulthood [3]. Treatments for FAs have included surgical excision, curettage, dermabrasion, laser ablation, pulse dye laser (PDL), electrocautery, cryosurgery, and topical rapamycin [4–10]. Nowadays, no guidelines or recommendations on the management of FAs are available, and lesser information exists for other TS skin cancers like shagreen patch [11–13].

The use of mTOR inhibitors like sirolimus (rapamycin) has significantly improved the treatment of TS cutaneous symptoms [14, 15]. However, topical sirolimus rarely results in complete treatment of angiofibromas. Indeed, the adult patients with fully grown angiofibromas respond less favourably to topical rapamycin treatment as demonstrated by Park et al. [8] who showed that the application of topical rapamycin was ineffective in the treatment of papules larger than 4 mm. In this instance, the large FAs can be effectively treated with rapamycin combined to laser therapy [16].

On the other hand, the cryotherapy or shave excision followed by dermabrasion is a simple procedure to use, but it presents various drawbacks, such as postoperative discomfort and hypopigmented scarring in susceptible patients [17].

As a result, physical treatment to diminish the size of both old and advanced lesions, especially for individuals who did not receive early care or who have had the condition for a long time, has been widely employed.

Laser therapy is considered an effective treatment for angiofibromas [18].

The existing literature already reported clinical cases in which the ablative and nonablative laser combination technique has led to good results in the resolution of TS-related angiofibroma lesions [19]. Especially, the combination of PDL and CO_2_ laser was successfully used for vascular lesions management [20, 21].

Herein, we discuss our experience, highlighting a rare occurrence of multiple FAs in young patient, successfully treated with ablative CO_2_ laser combined with PDL therapy.

## 2. Case Presentation

We report a clinical case of a 23-year-old male patient affected by tuberous sclerosis (TS) who manifests neurological symptoms represented by a mild mental retardation. Patient presents multiple erythematous and colored papules/nodules and raised erythematous elevated plaque located on the face, mainly concentrated around nose, perinasal area, cheeks, and chin area. The multiple papules measured up to 0.5 cm in size. The onset of TS symptoms occurred in adolescents, immediately prior to puberty. TS was confirmed by brain magnetic resonance imaging. Prior to the study, the patient did not receive any pharmaceutical therapies.

Since redness and volume of the lesions were the main cause of patient's cosmetic problems, a combinational laser therapy, including the use of the ablative CO_2_ laser (TetraPro—DEKA M.E.L.A, Calenzano, Italy) and a pulsed dye laser (Synchro VasQ—DEKA M.E.L.A, Calenzano, Italy), was administered.

The patient underwent 3 sessions of combined treatment with CO_2_ and pulsed dye laser.

The intralesional dye laser treatment was administered immediately after the CO_2_ laser session. The time interval between the combined laser treatments was approximately two months. A topic occlusive anaesthetic mask was applied 20 minutes before treatment with CO_2_ laser.

**Figure 1:**
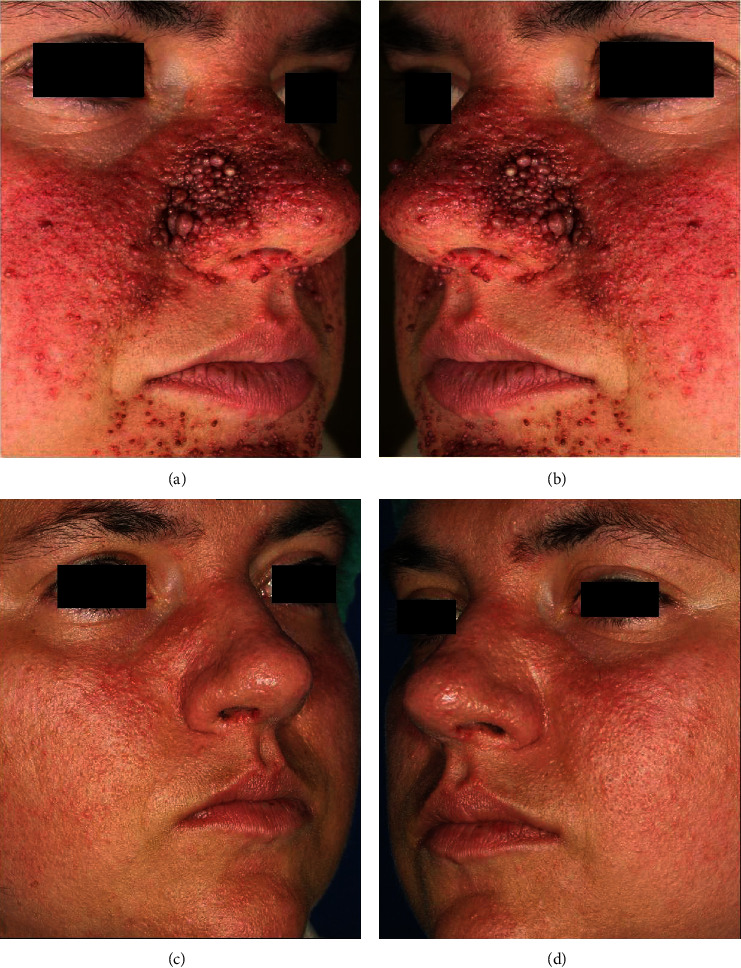
(a, b) Right and left lateral views of FAs located on the face of the young male patient. (c, d) Right and left lateral views of the same male patient at 4 months of follow-up after the last laser treatment session. A marked improvement of skin lesions was observed in all treated area.

For CO_2_ laser therapy, parameters were set to frequency 10 Hz, power 0.4-1.5 W, SP pulse. PDL was performed immediately after CO_2_ laser, and parameters of spot size 10–12 mm and fluence 7 J/cm^2^ were selected. A clinical photography-based assessment of the Facial Angiofibromata Severity Index (FASI) was used to determine the effectiveness of the treatment at the baseline and four months following the final laser treatment session. The FASI scores range from 0 (the best) to 3 (the worst), encompassing erythema (0–3), size (0–3), and extent (0, 2, or 3). In addition, the Dermatology Life Quality Index (DLQI) before and four months following the final laser treatment session was administered to the patient in order to assess the patient's level of satisfaction. The final DLQI score ranges from 0 (no impact on quality of life) to 30 (maximum impairment). Following combined laser therapy, most of the facial angiofibromas have shown remarkable improvement in redness and size. After 4 months from the last laser treatment session, most of the facial erythematous and protruding lesions had improved, as clearly shown in Figure 1. The PDL laser treatment interval was extended to a monthly frequency of 6 months as a maintenance therapy to avoid the reappearance of angiofibromas.

**Figure 2:**
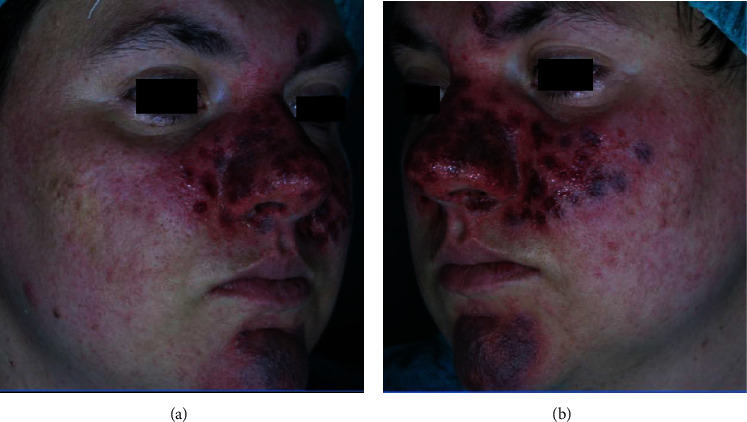
(a, b) Right and left lateral views of the young male patient immediately after dye laser session.

Following these procedures, the patient did not experience complications or severe adverse reaction. Intense purpura on the face was observed immediately after the dye laser procedure (Figure 2), but these conditions considerably disappeared after a week.

FASI indicates that all variables (redness, size, and extension) in our clinical image were considerably improved. The FASI score decreased from 9 at the baseline to 0 at four months of follow-up from the last laser treatment session. DLQI scores varied from 25 at the baseline to 2 at four months follow-up from the last laser treatment session indicating a marked impairment on patient's quality of life following the laser therapy.

At postoperative therapy, healing creams based on hyaluronic acid and an antibiotic cream were applied to the patient's face after careful cleaning of the wounds with gauze and saline solution. During and after the research study period, patients were also instructed to limit further photodamage by using sunscreen with a 50-sun protection factor.

In conjunction with maintenance treatment with dye laser, the use of topical rapamycin can be recommended to the patient in order to avoid the return of the vascular symptoms of angiofibromas.

## 3. Discussion

A laser combination therapy may be required for patients affected by angiofibromas [9]. Multiple laser treatment has been shown to reduce the risk of dyspigmentation and scarring while maximizing the therapeutic efficacy of each modality [13]. Among these, the combination of PDL laser and the CO_2_ laser was successfully tested [2].

PDL by targeting the vascular components of the FAs lesions, it is able to improve erythema in more than 90% of patients with predominant vascular components but without decreasing the fibrous component [9]. For this purpose, the ablative CO_2_ laser therapy has been used for its effectiveness in reducing the size of the fibrous component compared with PDL. Indeed, elevated lesions have been flattened using the CO_2_ laser since it is the best option for accurate, safe ablation with good hemostasis; by creating a matrix-shaped microthermal damage, fractional CO_2_ lasers stimulate the basic repair process in the dermis and in the epidermis, leading to skin regeneration and repairing.

The present case report confirms the effectiveness of this combined laser technique where the global improvement of the functional and aesthetic patient-reported outcomes was done by the CO_2_ healing process through excision and tissue regeneration and the action of the nonablative 595 nm laser, which provides more control over the healing process.

Our findings showed a great improvement of patient's skin lesions, and the results were evident immediately after the first treatment. All erythematous and prominent papules have exhibited notable improvements in size and redness at four months after the last laser therapy session as documented by clinical images and FASI results.

According to DLQI scores, the results achieved after treatment at the follow-up revealed that the condition has completely lost its negative impact on the daily lives of the patient. Furthermore, the patient did not experience complications or recurrence.

Usually, the rapamycin may have had an additive effect in lesion regression and maintenance due to its antiangiogenic activity and by blocking proliferation of abnormal fibroblasts [22]. However, prospective research including 25 TS patients showed that topical rapamycin cream maintenance treatment administered three times a week was ineffective in preventing TSC recurrence [23]. Consequently, in our study, as a primary choice, we favoured the use of dye laser as a maintenance therapy to avoid the reappearance of FAs. However, under medical evaluation, in conjunction with maintenance treatment with dye laser, the topical rapamycin can be administered.

In contrast in the study of Neamonitou et al. [24], patients who manifest recurrence were retreated with CO_2_ laser. This research was not in line with our study protocol as the subjects received a triple laser therapy (CO_2_ laser, erbium laser, and PDL) in one session, achieving good results. However, the CO_2_ laser employed in our research offers an innovative technology which permits users to simulate the Er-YAG laser effect by modulating the power and pulse parameters. In this way, we can reduce the coagulative effect in order to obtain a superficial skin resurfacing and a reduction of skin lesion thickness. It represents a noninvasive and not painful technology that did not require the use of anaesthesia, which is instead used in other studies [24, 25]; this makes the system also suitable to treat individuals who exhibit neurological signs, such as our clinical case.

A multiple combined session of different lasers was also used in the study of Fioramonti et al. [17] where all patients showed great improvement of their skin lesions with no complications or recurrence.

The absence of skin biopsy specimen examination of the face papules and a longer follow-up period in order to monitor the recurrence rate were the primary study limitations. In conclusion, the combined use of the CO_2_ and dye laser has been proved to be a safe and effective treatment for multiple FAs in the young patient affected by tuberous sclerosis.

## Data Availability

The data that support the findings of this study are available from the corresponding author upon reasonable request.

## References

[1] Boggarapu S., Roberds S. L., Nakagawa J., Beresford E. (2022). Characterization and management of facial angiofibroma related to tuberous sclerosis complex in the United States: Retrospective analysis of the natural history database. *Orphanet Journal of Rare Diseases*.

[2] Weiss E. T., Geronemus R. G. (2010). New technique using combined pulsed dye laser and fractional resurfacing for treating facial angiofibromas in tuberous sclerosis. *Lasers in Surgery and Medicine*.

[3] Zöllner J. P., Franz D. N., Hertzberg C. (2020). A systematic review on the burden of illness in individuals with tuberous sclerosis complex (TSC). *Orphanet Journal of Rare Diseases*.

[4] Fischer K., Blain B., Zhang F., Richards L., Lineaweaver W. C. (2001). Treatment of facial angiofibromas of tuberous sclerosis by shave excision and dermabrasion in a dark-skinned patient. *Annals of Plastic Surgery*.

[5] Capurro S., Fiallo P. (2001). Timed surgery for treatment of angiofibromas in tuberous sclerosis. *Dermatologic Surgery*.

[6] Truchuelo T., Dı´az-Ley B., Rı´os L., Alca´ntara J., Jae´n P. (2012). Facial angiofibromas treated with topical rapamycin: An excellent choice with fast response. *Dermatology Online Journal*.

[7] Salido-Vallejo R., Garnacho-Saucedo G., Moreno-Giménez J. C. (2014). Current options for the treatment of facial angiofibromas. *Actas Dermo-Sifiliográficas*.

[8] Park J., Yun S. K., Cho Y. S., Song K. H., Kim H. U. (2014). Treatment of angiofibromas in tuberous sclerosis complex: The effect of topical rapamycin and concomitant laser therapy. *Dermatology*.

[9] Papadavid E., Markey A., Bellaney G., Walker N. P. (2002). Carbon dioxide and pulsed dye laser treatment of angiofibromas in 29 patients with tuberous sclerosis. *British Journal of Dermatology*.

[10] Wheless J. W., Almoazen H. (2013). A novel topical rapamycin cream for the treatment of facial angiofibromas in tuberous sclerosis complex. *Journal of Child Neurology*.

[11] Seo Ji Y., Kim A., Baek Y. S., Jeon J. (2023). Successful long-term multimodality management of facial lesions in tuberous sclerosis complex in an adult patient. *Annals of Dermatology*.

[12] Wang B., Yao Y., Huang X., Zhang L., Peng D., Zhang G. (2022). The combination of photodynamic therapy and ultrapulse carbon dioxide laser for facial angiofibromas in tuberous sclerosis complex: A case report. *Photodiagnosis and Photodynamic Therapy*.

[13] Bae-Harboe Y. S., Geronemus R. G. (2013). Targeted topical and combination laser surgery for the treatment of angiofibromas. *Lasers in Surgery and Medicine*.

[14] Balestri R., Rizzoli L., Pedrolli A. (2023). Analysis of current data on the use of topical mTOR inhibitors in the treatment of facial angiofibromas in tuberous sclerosis complex-An update. *Journal of the European Academy of Dermatology and Venereology*.

[15] Koenig M. K., Bell C. S., Hebert A. A. (2018). Efficacy and safety of topical rapamycin in patients with facial angiofibromas secondary to tuberous sclerosis complex: The TREATMENT randomized clinical trial. *JAMA Dermatology*.

[16] Negosanti F., Tengattini V., Gurioli C., Neri I. (2018). Facial angiofibromas treated by rapamycin 0.05% ointment and a combined laser therapy. *Journal of Cosmetic Dermatology*.

[17] Fioramonti P., De Santo L., Ruggieri M. (2014). CO_2_/Erbium: YAG/Dye laser combination: An effective and successful treatment for angiofibromas in tuberous sclerosis. *Aesthetic Plastic Surgery*.

[18] Campolmi P., Bonan P., Cannarozzo G. (2003). Laser e sorgenti luminose in dermatologia. *Masson*.

[19] Gu Y., Verheyden M. J., Sebaratnam D. F., Liu R. C. (2024). A systematic review of laser treatment for angiofibromas in tuberous sclerosis. *Dermatologic Surgery*.

[20] Lodi G., Sannino M., Cannarozzo G., Bennardo L., Nisticò S. P. (2022). Pulsed dye laser prior to 2 laser ablation to treat multiple cutaneous neurofibromas in von Recklinghausen’s disease: A case report. *Dermatologic Therapy*.

[21] Sannino M., Ambrosio A. G., Lodi G., Cannarozzo G., Bennardo L., Nisticò S. P. (2021). A giant epidermal nevus of the face treated with a CO2 and dye laser combination: A case report and literature review. *Journal of Cosmetic and Laser Therapy*.

[22] Oh J., Kim J., Lee W. J., Lee J. H. (2019). Use of topical rapamycin as maintenance treatment after a single session of fractionated CO2 laser ablation: A method to enhance percutaneous drug delivery. *Annals of Dermatology*.

[23] Malissen N., Vergely L., Simon M., Roubertie A., Malinge M. C., Bessis D. (2017). Long-term treatment of cutaneous manifestations of tuberous sclerosis complex with topical 1% sirolimus cream: A prospective study of 25 patients. *Journal of the American Academy of Dermatology*.

[24] Neamonitou F., Neamonitos K. K., Stavrianos S., Neamonitos K. P. (2024). A triple laser combination treatment for facial Angiofibromata management in tuberous sclerosis and literature review. *Arch Plast Surg*.

[25] Gu Y., Verheyden M. J., Sebaratnam D. F., Liu R. C. (2024). A systematic review of laser treatment for angiofibromas in tuberous sclerosis. *Dermatologic Surgery*.

